# Social Network Forensics Analysis Model Based on Network Representation Learning

**DOI:** 10.3390/e26070579

**Published:** 2024-07-07

**Authors:** Kuo Zhao, Huajian Zhang, Jiaxin Li, Qifu Pan, Li Lai, Yike Nie, Zhongfei Zhang

**Affiliations:** 1 School of Intelligent Systems Science and Engineering, Jinan University, Zhuhai 519070, China; zhjjnu@stu2021.jnu.edu.cn (H.Z.); panqf@stu2022.jnu.edu.cn (Q.P.); fwhirlwind3@gmail.com (L.L.); nykjndx@stu2022.jnu.edu.cn (Y.N.); 2 Guangdong International Cooperation Base of Science and Technology for GBA Smart Logistics, Jinan University, Zhuhai 519070, China; 3 Institute of Physical Internet, Jinan University, Zhuhai 519070, China; 4 School of Management, Jinan University, Guangzhou 510632, China

**Keywords:** network representation learning, social network forensics, node vectorization, node2vec algorithm, gradient update, hierarchical clustering

## Abstract

The rapid evolution of computer technology and social networks has led to massive data generation through interpersonal communications, necessitating improved methods for information mining and relational analysis in areas such as criminal activity. This paper introduces a Social Network Forensic Analysis model that employs network representation learning to identify and analyze key figures within criminal networks, including leadership structures. The model incorporates traditional web forensics and community algorithms, utilizing concepts such as centrality and similarity measures and integrating the Deepwalk, Line, and Node2vec algorithms to map criminal networks into vector spaces. This maintains node features and structural information that are crucial for the relational analysis. The model refines node relationships through modified random walk sampling, using BFS and DFS, and employs a Continuous Bag-of-Words with Hierarchical Softmax for node vectorization, optimizing the value distribution via the Huffman tree. Hierarchical clustering and distance measures (cosine and Euclidean) were used to identify the key nodes and establish a hierarchy of influence. The findings demonstrate the effectiveness of the model in accurately vectorizing nodes, enhancing inter-node relationship precision, and optimizing clustering, thereby advancing the tools for combating complex criminal networks.

## 1. Introduction

The proliferation of mobile internet technology has significantly enhanced communication across various platforms, including phone calls, emails, and social media networks such as WeChat and Weibo. By the end of 2023, China’s Ministry of Industry and Information Technology reported nearly 1.727 billion mobile phone users, and WeChat’s monthly active accounts surpassed 1.327 billion. The convenience, speed, and immediacy of mobile networks have rendered online social interactions indispensable in both personal and professional spheres. Originating from email, social networks have evolved to fulfill a variety of social requirements, resulting in the accumulation of valuable Mobile Communication Data (MCD) and Criminal Incident Reports (CIRs) [1,2,3]. These datasets not only reveal personal relationships but are also instrumental in social network forensics.

In social network forensics, the Social Network Forensic Analysis (SNFA) model is utilized to detect important actors within criminal networks via email interactions, potentially identifying leaders or suspects based on their centrality. This application of the SNFA model highlights its importance in aiding law enforcement agencies in their investigative and judicial endeavors by providing a means to analyze the structure of criminal networks and pinpoint key participants.

However, it is important to note that applying the SNFA model to modern social network apps or communication platforms such as WeChat might not yield effective results due to the presence of irrelevant content and tenuous connections. These platforms often contain vast amounts of non-criminal-related interactions, which can dilute the forensic relevance of the data. This limitation underscores the necessity of carefully selecting and preprocessing data to ensure their applicability in forensic investigations.

Challenges arise owing to the sheer volume and authenticity of interactive data, as well as the heterogeneity of users and information [4,5]. Analyzing MCD and CIRs is crucial for comprehending the structure of criminal networks and pinpointing key participants, thereby aiding law enforcement agencies in their investigative and judicial endeavors [6]. In social network forensics, mobile data reveals user connections and communication content. Forensic methods are evolving with technology, focusing on network data, such as logs and streams, often through real-time capture using sniffing technologies.

Research on web-based forensics predominantly encompasses the analysis of dynamic, real-time information across mobile networks, leveraging sniffing technologies to glean and secure network data flows. In the realm of evidentiary representation, Erbacher, R.F. and Christensen, K. introduced an innovative forensic visualization paradigm in 2006 [7], which enhanced the application of forensic technology in actual crime scenes. Network forensics techniques can be categorized into server-based, client-based, and data-flow-based methodologies. Although the advent of cloud computing has complicated access to server-side data, suggesting limitations to server-based forensics, client-based techniques continue to offer the thorough extraction and analysis of individual computer records. Meanwhile, data-flow forensics provide a holistic snapshot of user activity. However, forensic investigation of web-based social networks is not without challenges, given the security measures of platforms and user privacy controls.

In semantic forensic analysis, Natural Language Processing (NLP) [8] tools are pivotal in identifying incriminating evidence within textual content [9,10]. Foundational NLP models, such as Latent Semantic Analysis (LSA) [11,12], Probabilistic Latent Semantic Analysis (PLSA), and Latent Dirichlet Allocation (LDA) [13,14] serve to reduce noise and unearth thematic structures, whereas algorithms such as TF-IDF [15], BM25 [16], and TextRank [17] play a crucial role in keyword extraction. Techniques such as Hidden Markov Models (HMMs) [18] and Conditional Random Fields (CRFs) [19] are integral to lexical scrutiny. These methodologies enhance texts with poor semantic frameworks, laying the groundwork for advanced NLP tasks, including machine translation and sentiment analysis.

Community detection algorithms trace their origins to early algorithms such as Girvan and Newman’s GN algorithm [20] and its refined version, the Fast Newman algorithm [21], which addresses longstanding issues in identifying intricate network relationships, often exacerbated by challenges such as community overlap [22,23] and dynamic network [24,25] evolution. Cutting-edge solutions, such as the Louvain Algorithm [19], Infomap [26], the Label Propagation Algorithm (LPA) [27], and the Girvan–Newman Algorithm [28], have substantially improved the process of community detection. These instruments are instrumental in identifying denser nodes within a network, shedding light on complex structures and their evolution. They utilize distinct approaches and metrics, such as modularity, to increase the accuracy of community delineation. Nevertheless, the analysis of multilayer networks remains intricate because of the complex interconnections across layers, which calls for more sophisticated analytical tools.

Advancements in the computational power and proliferation of social networks have transformed social network forensics into an essential field of research. The aforementioned methods, limited to the analyses of single-layer networks, often neglect the complexity of the node characteristics and indirect relationships [29]. Traditional forensic methods tend to overlook the multi-dimensional nature of social networks, focusing primarily on surface-level interactions and failing to capture the full extent of relationships within the network. To overcome these limitations, our study introduces a multidimensional network representation learning approach [30] that captures both direct and indirect node connections.

The proposed SNFA model, an advanced hierarchical system, integrates network attributes such as small-world phenomena and scale-free properties to enhance node evaluation. By combining Node2vec [31] with the Continuous Bag-of-Words (CBOW) approach [32] and hierarchical Softmax [33], the model fine-tunes the node sampling and gradient optimization processes that correspond to the inherent stratification of networks. Furthermore, hierarchical clustering techniques are utilized to identify key nodes, significantly improving the identification of pivotal elements within criminal networks. Innovations of the SNFA model include (1) employing network representation learning to represent criminal networks in vector space, streamlining the analysis of node relationships, inclusive of those among non-adjacent nodes; (2) enhancing the random walk sampling procedure and gradient update mechanism in the CBOW model’s output layer via Node2vec; and (3) leveraging hierarchical clustering to achieve comprehensive clustering structures and identify optimal central values, using diverse distance metrics to determine the significance of nodes at the core of the network.

Here are the major contributions of this paper:

1. Introduction of SNFA Model: This paper introduces the SNFA model, which employs network representation learning to identify and analyze key figures within criminal networks, including leadership structures.
2. Utilization of Network Representation Learning: The model incorporates traditional web forensics and community algorithms, utilizing concepts such as centrality and similarity measures, and integrating algorithms such as Deepwalk, Line, and Node2vec to map criminal networks into vector spaces while maintaining node features and structural information.
3. Innovative Node Relationship Refinement: The model refines node relationships through modified random walk sampling using BFS and DFS and employs a Continuous Bag-of-Words with Hierarchical Softmax for node vectorization, optimizing value distribution via the Huffman tree.
4. Enhanced Forensic Analysis Techniques: By leveraging hierarchical clustering and distance measures (cosine and Euclidean), the model identifies the key nodes and establishes a hierarchy of influence. This approach demonstrates the model’s effectiveness in accurately vectorizing nodes, enhancing inter-node relationship precision, and optimizing clustering.
5. Application and Evaluation: The application of the model to Enron emails serves as a case study to compare metrics with two other models, highlighting its practical utility in forensic investigations.

In the subsequent sections of this paper, we delve into various facets of the Social Network Forensic Analysis (SNFA) model to elaborate on its framework and functionality. Section 2, ‘Materials and Methods’, provides an in-depth examination of the model architecture, focusing on the selection and analysis of nodes within social networks and the computational strategies employed to enhance forensic analysis accuracy. Section 3, ‘Results and Discussion’, presents the outcomes of our empirical tests, demonstrating the efficacy of the SNFA model through a comparative analysis with existing forensic approaches. Finally, Section 4, ‘Conclusions and Outlook’, summarizes the contributions of this research and discusses potential avenues for further enhancements and applications of the SNFA model in the field of digital forensics. Through these discussions, we aim to illustrate the robustness of our approach in improving the precision and scalability of social network forensics.

## 2. Materials and Methods

In this section, we introduce the SNFA model, a social network forensic analysis framework based on representation learning methods. Due to the characteristics of social networks such as scale-freeness, small-world phenomena, community structures, and hierarchical nature, SNFA constructs a hierarchical network model to identify key nodes. The following sections analyze several aspects: the selection of computable nodes in the network, improvements in the sampling encoding model and random walk strategies, gradient computation, layered clustering division of the network, and acquisition and calculation of the network’s core nodes.

### 2.1. Enhanced Node Sampling Precision

The accuracy of node sampling within the realm of social network forensics is essential for precise identification and analysis of key nodes in criminal networks. The complexity and vastness of social networks require improved sampling accuracy to efficiently identify the nodes of interest, reduce computational demands, and enhance the effectiveness of forensic analyses. This section will present the algorithmic enhancements integrated into our Social Network Forensic Analysis (SNFA) model that aim to refine node sampling accuracy. Essential to uncovering the structure of criminal activities, these advancements facilitate the accurate identification of critical nodes in forensic analysis. We explain the improvements to our node sampling methodology, covering both theoretical foundations and practical applications, to offer a detailed understanding of our contribution to network forensics. The success of social network forensic analysis largely depends on the node sampling precision, which impacts the identification accuracy of key nodes in criminal networks. While traditional methods are broadly effective, they often lack the required specificity for forensic analysis, where pinpointing behaviorally significant nodes is paramount. We have thus significantly enhanced the Node2vec algorithm, a key component of our SNFA model, and developed new sampling strategies to improve precision.

1. Modifications to Node2vec:

Our modified Node2vec algorithm incorporates a weighted random walk mechanism that prioritizes nodes based on their forensic relevance, diverging from the traditional approach that treats all nodes with equal importance during the walk. By integrating forensic relevance as a criterion for node selection, our approach ensures that nodes with higher potential forensic values are sampled more frequently, thus increasing the focus of the model on areas of the network that are most likely to yield valuable insights.

Mathematically, we introduce a weighting function, $W\left(n\right)$, which assigns a forensic relevance score to each node, n, based on predefined criteria, such as node centrality, frequency of communication, and anomalous behavior patterns. The probability of transitioning from node i to node j during a random walk is then adjusted as follows:

(1) $$P\left(i\to j\right)=\frac{\alpha \cdot W\left(j\right)}{\sum_{k\epsilon N\left(i\right)}W\left(k\right)},$$

where α is a normalization factor ensuring that the probabilities sum to 1 and $N\left(i\right)$ represents the set of neighbors of node i.

2. Novel Sampling Strategies:

Beyond the modifications to Node2vec, we introduce a novel sampling strategy that further refines the selection of nodes for analysis. This strategy employs a two-tiered approach, initially segmenting the network into clusters based on structural and behavioral similarities, and subsequently applying our enhanced Node2vec algorithm within each cluster. This approach allows for more focused sampling within areas of the network that are homogenous in terms of forensic characteristics, thereby improving the overall precision of node sampling.

To operationalize these improvements, we present the following pseudocode, illustrating the modified Node2vec algorithm with an integrated weighting function for forensic relevance:

Algorithm 1 marks a pivotal enhancement in our model, integrating forensic relevance to refine the random walk process for social network forensic analysis. This modification of the Node2vec algorithm, as outlined in Algorithm 1, highlights the crucial role of domain-specific insights in algorithmic development. By assigning priority to nodes based on forensic significance, we enhanced the precision of identifying crucial network nodes and boosted investigative efficiency and depth. This advancement aligns with our Social Network Forensic Analysis (SNFA) model’s goal of offering a focused, accurate, and comprehensive tool for dissecting criminal activity in complex networks. This incorporation of forensic relevance is set to enrich forensic analysis and open new investigative pathways in the dynamic field of digital forensics.
**Algorithm 1.** Enhanced Node2vec.Input: Graph G(V, E), Weighting function $W\left(n\right)$, Walk length L, Start node s Output: Sequence of nodes S representing a weighted random walk
Initialize sequence S with start node sfor i=1 to L do2.1Let c be the current node (last node in S)2.2Calculate total weight T=sum(W(n) for n in neighbors(c))2.3For each neighbor n of c, calculate transition probability P(c → n)=(W(n)/T)2.4Select next node n′ based on transition probabilities P(c → n)2.5Append n′ to sequence S
return S

### 2.2. Selection of Computable Nodes

In the complex domain of social network forensics, the scale-free nature of networks means that many nodes have low degrees and their connections do not carry significant information [34]. Including these nodes with minimal informational content not only fails to improve experimental outcomes but also increases computational time and algorithm complexity.

Within these networks, certain nodes may be suspicious, thus introducing the concepts of node synchronization and anomalies. Synchronization refers to the high similarity and parallel behaviors among nodes, where excessive synchronization can compromise the experimental accuracy. Anomaly, on the other hand, indicates a node’s deviation in behavior from the majority, reducing the accuracy if such nodes are dispersed within the network graph.

Moreover, incorporating numerous low-degree nodes can invalidate sampling the parameters in algorithms such as Node2vec, which uses parameters p and q to balance the Breadth-First Search (BFS) [35] and Depth-First Search (DFS) [36] in random walks. If a node’s degree is too low, adjusting these parameters does not alter the random walk pattern, as exemplified in social networks where a node with a single connection always leads to the same random walk sequence, regardless of p and q values.

To circumvent these issues, rules for selecting computable nodes and eliminating low degree, synchronized, or anomalous nodes from the network are necessary for more efficient and accurate computations. The experimental criteria for computable nodes include the following:

1. Formulas for synchronization and normalization were utilized to filter out synchronized and anomalous nodes in the network [37]. Node u’s synchronization is defined by the similarity between every pair of nodes, as shown in Equation (2), and its normalization by the average similarity with the majority of the other nodes, as in Equation (3). Here, $c(v,v^{\prime })$ represents the similarity between nodes v and $v^{\prime }$, $O\left(u\right)$ is the set of target nodes for node u, $d_{o}\left(u\right)$ is the count of outward nodes, i.e., the size of $O\left(u\right)$, and N is the total number of nodes. These metrics are influenced by connected nodes, akin to the PageRank algorithm.
  (2) $$sync\left(u\right)=\frac{\sum_{\left(v,v^{\prime }\right)\in O\left(u\right)*O\left(u\right)}c\left(v,v^{\prime }\right)}{d_{o}\left(u\right)*d_{o}\left(u\right)}$$
  (3) $$norm\left(u\right)=\frac{\sum_{\left(v,v^{\prime }\right)\in O\left(u\right)*u}c\left(v,v^{\prime }\right)}{d_{o}\left(u\right)*N}$$
2. Selecting nodes in the network with a degree greater than 1.
3. The total communication count of a node divided by the number of nodes it communicates with should exceed the average total communication count per node across the network. The selection rule is as follows:
  (4) $$\left\{\begin{array}{c} \frac{W\left(w\right)}{P\left(w\right)}\geq \frac{\sum_{i=1}^{N}W\left(i\right)}{\sum_{i=1}^{N}P\left(i\right)}i,u\in G,N=\left|G\right|\\ P\left(u\right)>1 \end{array}\right..$$

### 2.3. Node Sampling and Encoding

In social network analysis, node sampling and encoding are critical for converting nodes into computational representations. The Node2vec algorithm [31], based on the foundation of Word2vec [38,39] from natural language processing, improves the adaptation for analyzing network structures. It treats nodes in a manner akin to words in natural language and undergoes a sampling process to represent their interrelations and properties.

Random walks across the network generate “sentences” of nodes as part of the sampling phase, reflecting the contextual background of each node. The frequency of sampling and depth of the walks are integral to defining the degree of connection between nodes, with more frequently sampled nodes suggesting closer ties. Node2vec’s parameters, p and q, modulate the sampling between breadth-first and depth-first explorations, enhancing the detection of less direct relationships.

Encoding succeeds in sampling and translating the nodes into computer-readable formats. Although one-hot encoding is straightforward, it becomes unfeasible for larger networks owing to scalability issues. Vector encoding methods [40], central to Deepwalk [41], Word2vec, and Node2vec, address this by mapping each node to a dense, low-dimensional vector, reducing the computational load and better capturing nodal features. This vector space model aids in machine learning, allowing quantitative analysis of node relationships and similarities.

Tomas Mikolov introduced two formative models in 2013, Skip-gram and Continuous Bag-of-Words (CBOW) [42], applying neural network approaches for effective word vector encodings. These models accelerate training using simplified architectures and underpin statistical language modeling techniques.

The CBOW model predicts the probability of a node from the context of adjacent nodes and consists of three layers: output, projection, and input. Given a target node, $w_{i}$, within its context, represented as $\left\{W_{i-c},\ldots ,W_{i-1},W_{i},W_{i+1},\ldots ,W_{i+c}\right\}$, the CBOW constructs a context set from the surrounding nodes. The model endeavors to maximize its likelihood function, $P=p(w|Context\left(w\right))$, across all nodes N.

**Figure 1:**
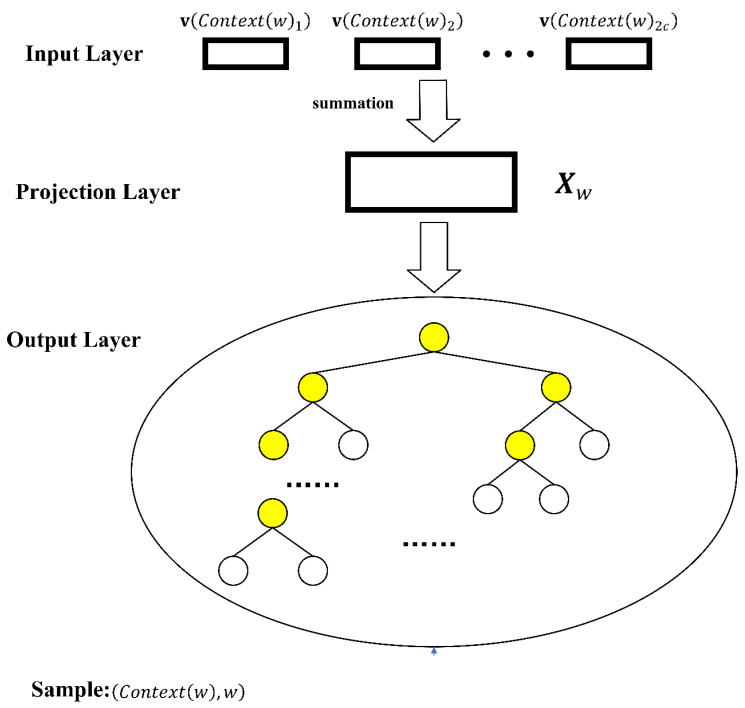
CBOW model structure.

CBOW’s three-tier architecture is depicted in Figure 1.

1. The input layer processes 2c neighboring nodes around $w_{i}$ as vectors, with the window size c set to 2.
2. The projection layer aggregates input vectors into a summary vector.
3. The output layer forms a Huffman tree, whereby terminal nodes align with network nodes and non-terminal nodes embody vector representations. In the diagram, non-terminal nodes are highlighted in yellow to indicate their role in the hierarchical structure of the tree, as they are responsible for the intermediate calculations in the encoding process, while the terminal nodes are not highlighted as they represent the final output in the Huffman tree structure.

In contrast to CBOW, the Skip-gram model infers the surrounding nodes from the vector encoding of a single node. Both models employ Negative Sampling [43] and Hierarchical Softmax, with the CBOW model’s optimization aiming to condense the Huffman tree encodings for efficiency and encoding fidelity.

### 2.4. Constructing the SNFA Forensic Model

While constructing the SNFA forensic model, two crucial concepts are introduced to reflect the key roles of individuals, or nodes, within social networks: activity and significance. The activity level, denoted as $W\left(u\right)$, indicates the communication frequency of node u, with higher values representing more active nodes. Forensic significance, expressed as $imp\left(u\right)=P\left(u\right)W\left(u\right)$, combines the unique number of communicating nodes, $P\left(u\right)$, with the total communication count, $W\left(u\right)$, to gauge the importance of a node within the network.

Utilizing vectors for representation, the SNFA model employs the Node2vec algorithm, initially based on the CBOW model, to capture the importance of the nodes. Social networks are modeled as undirected weighted graphs, where individuals are nodes and communication frequencies form edge weights. Node2vec’s random walk mechanism, adjusted by parameters p and q, navigates the trade-off between exploring local and global network structures. However, this adjustment is network-wide and overlooks the nuances of the individual node relationships. The probability of transitioning between two nodes is weighted according to their relative communication frequency, with respect to the total communications initiated by the starting node, and is formalized as follows:

(5) $$k=\frac{w_{t,x}}{W\left(t\right)}\;t\in E,$$

with $w_{t,x}$ being edge $\left(t,x\right)$ in the network.

The transition probability from one source node to another within a communication network is defined as follows:

(6) $$\left(c_{i}=x|c_{i-1}=v\right)=\left\{\begin{array}{c} \frac{\pi_{vx}}{Z}\;\;if(v,x)\in E\\ 0\;\;\;\;\;otherwise \end{array}\right.,$$

with $\pi_{vx}$ being the unnormalized transition probability and Z a normalization constant.

**Figure 2:**
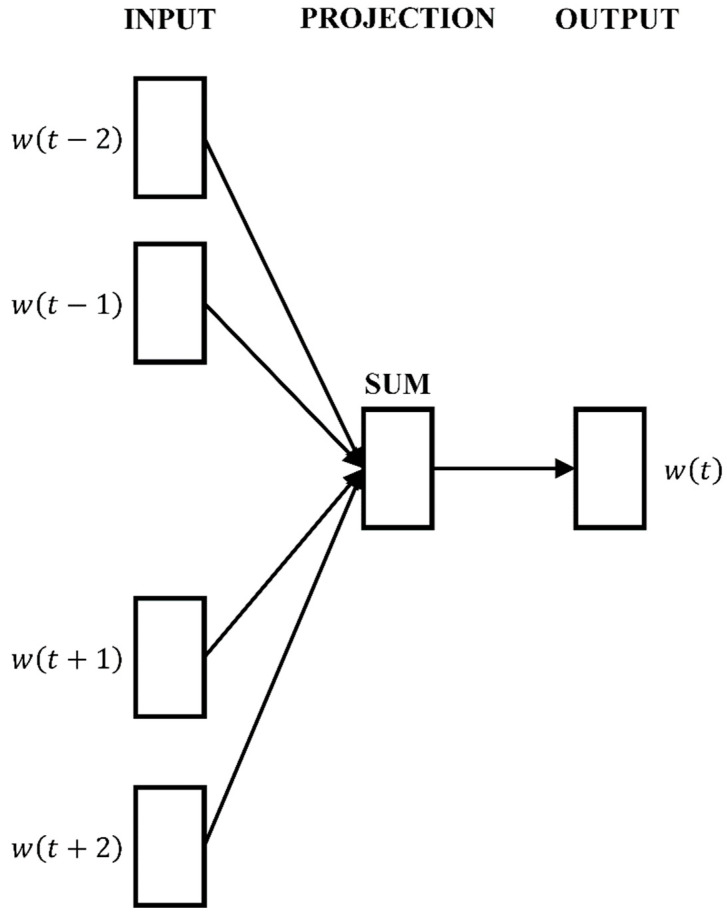
CBOW and Hierarchical Softmax model structure.

The SNFA model applies Node2vec for node sampling and encoding, representing social network *G*(*V*, *E*) nodes as vectors [41], which encapsulates node attributes and network structure. Given the extensive data, acceleration algorithms, such as Negative Sampling and Hierarchical Softmax, were employed, with the latter chosen for this study. The Hierarchical Softmax algorithm facilitates efficient vector updates, evenly distributing the gradient updates across all nodes to prevent the over-amplification of values. The structural diagram of the algorithm is shown in Figure 2.

This approach introduces the concept of average contribution by dividing the update value from each gradient ascent equally among nodes, aligning it more closely with the network’s structure and ensuring a balanced update across the network.

### 2.5. Utilization of Hierarchical Clustering and Distance Formulas

In the intricate task of analyzing criminal networks on social media, our Social Network Forensic Analysis (SNFA) model utilizes hierarchical clustering and distance formulas to improve the identification and ranking of core nodes. This section explains the application of these methodologies within the SNFA model and highlights their importance in forensic analysis.

The SNFA model makes use of Agglomerative Hierarchical Clustering (AHC), which is well-suited for dissecting the layered structures inherent in criminal networks. Unlike partitioning clustering methods that require a predefined number of clusters, AHC merges nodes or existing clusters iteratively, starting from individual nodes, thereby naturally adapting to the network’s structure. This bottom-up approach aids in uncovering nested communities that mirror the operational hierarchies commonly observed in criminal networks.

The benefit of employing AHC in our model lies in its capability to unveil subtle groupings within the network, which is essential for comprehending the roles and relationships among individuals involved in criminal activities. By constructing a dendrogram, AHC provides a visual and analytical framework for evaluating the connectivity and proximity of nodes, enabling forensic analysts to pinpoint pivotal nodes and their spheres of influence within the network.

To merge clusters effectively and identify core nodes, the SNFA model utilizes a combination of Euclidean distance and cosine similarity distance measures. Euclidean distance is crucial for measuring the direct dissimilarity between nodes, facilitating the initial clustering of closely connected individuals. This straightforward calculation renders it a reliable choice for identifying distinct subgroups within a network.

Cosine similarity, represented by Equation (7), measures the angle between the vector representations of two nodes and provides insights into the similarity in their interaction patterns rather than their magnitude. This metric is especially valuable in forensic scenarios where communication patterns, including frequency, timing, and common contacts, are more critical than the mere volume of communication.

(7) $$Cosine\;Similarity\left(n_{i},n_{j}\right)=\frac{\overset{⃑}{n_{i}}\cdot \overset{⃑}{n_{j}}}{\left\|\overset{⃑}{n_{i}}\right\|\left\|\overset{⃑}{n_{j}}\right\|}$$

By integrating these distance measures, the SNFA model identifies core nodes by evaluating their direct interactions and behavioral similarities, thereby enhancing the accuracy of the model in pinpointing individuals central to criminal activities.

The implementation of Agglomerative Hierarchical Clustering and sophisticated distance formulas within the SNFA model significantly improves our ability to dissect and comprehend criminal networks on social media. By meticulously clustering nodes based on their interactions and behavioral patterns, we enhanced the forensic analysis process, enabling more precise and detailed identification of core nodes. This approach aligns with the overarching objective of providing a comprehensive tool for social network forensics and offers a scalable and adaptable solution to the challenges posed by the intricate and dynamic nature of criminal networks.

### 2.6. Acquiring Key Figures in Social Network Forensics

In social network forensics, the SNFA model is utilized to detect important actors within criminal networks via email interactions, potentially identifying leaders or suspects based on their centrality. Core node identification assesses two key metrics: the frequency of communication, denoted by $W\left(u\right)$, and the network-wide extent of communication, expressed as follows:

(8) $$imp\left(u\right)=P\left(u\right)W\left(u\right).$$

Vector representation, adopted by the SNFA model through techniques such as Node2vec, is shaped by the community structure and hierarchical nature inherent to social networks. Clustering algorithms are then executed to determine the core nodes and rank their similarities to gauge their status in criminal networks.

Machine learning employs unsupervised learning for clustering when training samples are unlabeled. This approach categorizes samples into distinct clusters for pattern identification. Classic K-Means clustering iteratively groups samples into k clusters based on their centrality [44]. However, initial cluster center selection and outlier sensitivity can compromise K-Means accuracy, issues K-Means++ seeks to address through strategic initial center placements [45]. 

Considering the community and hierarchical structures of social networks, the SNFA model implements hierarchical clustering [46] to define an optimal cluster count, leveraging AGNES to merge the nearest clusters progressively using the average linkage criteria. Hierarchical clustering is notable for enabling a comprehensive one-time clustering execution, providing flexibility in cluster quantity [47]. 

Hierarchical clustering identifies a set of core nodes, N, of varying significance. The SNFA model correlates the importance of these nodes with sentencing data from the Enron corpus to discern leaders within the network. Vectors represent nodes and their interrelations, with vector similarity commonly measured by Minkowski, Euclidean, or Manhattan metrics [48]. However, a more nuanced node relationship analysis emerges when cosine similarity is combined with Euclidean distance.

The aggregated average cosine and Euclidean distances of the core nodes to others in N facilitate a multi-faceted importance assessment. The clustering pseudocode of the SNFA model specifies the steps for computable node selection, Node2vec encoding, and hierarchical clustering application for core node detection. This integration of distance measures refines the significance rankings, highlighting the key influencers of the network.

**Figure 3:**
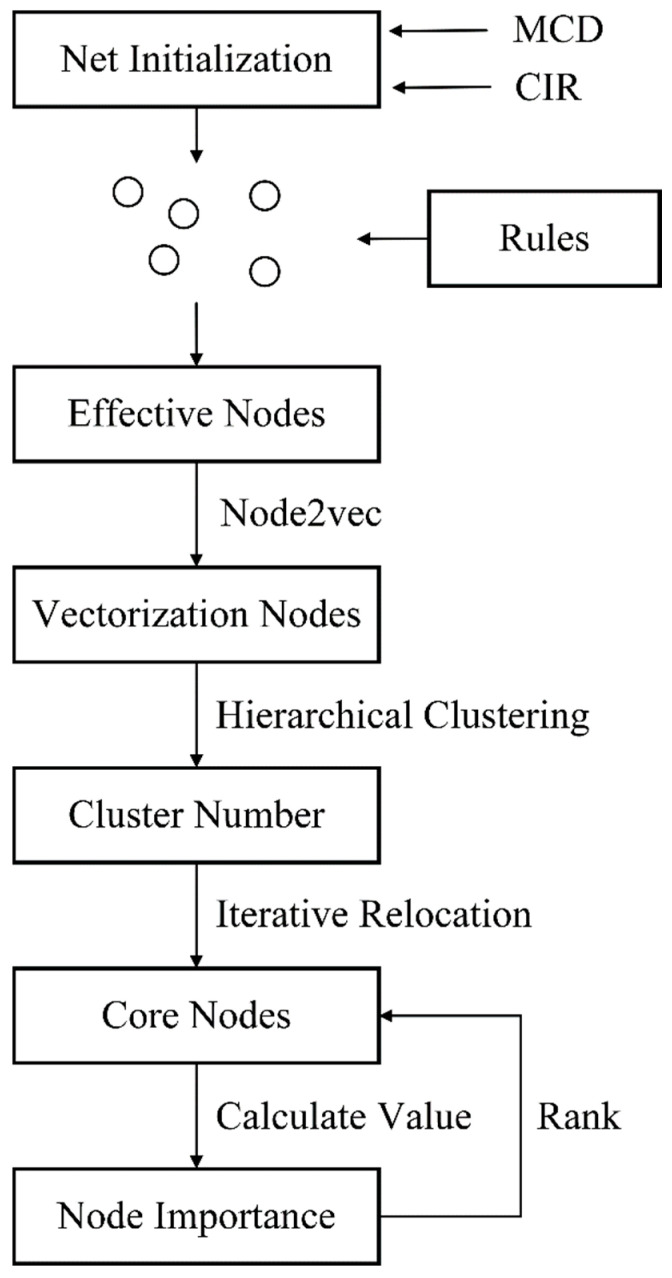
SNFA model structure.

The streamlined structure of the SNFA model, depicted in Figure 3, commences with social network initialization using either the MCD or CIR datasets. The selection of computable nodes adheres to established network principles, and Node2vec translates the criminal network into a vector space, optimizing both sampling and gradient computations. Hierarchical clustering then builds a complete dendrogram to identify the optimal cluster centroids. Through iterative refinement, the significance of core nodes is quantified and ordered, aiding in the identification of pivotal actors or suspects in criminal networks [49].

## 3. Experiment and Results

This section briefly outlines the experimental section of the SNFA model, describing the data background, data preprocessing, and experimental parameters of the model, as well as the final experimental results obtained. It also compares and analyzes these results against those from forensic models such as LogAnalysis and CrimeNet Explorer, measuring them against typical standards of accuracy, including recall, precision, and the F-measure, to demonstrate the construction and analytical capabilities of the SNFA model.

### 3.1. Experimental Procedure

Experimental data primarily take two forms: Mobile Communication Data (MCD) and Criminal Incident Reports (CIRs). Despite the wealth of information available from communications on platforms such as QQ, WeChat, Weibo, email, and Twitter, these data frequently include irrelevant content and tenuous connections, lacking robust evidence of criminal networks, and thus are unsuitable for the SNFA model. In light of considerations such as data relevance, structure, and volume, the SNFA model leverages Enron Corporation’s email dataset, a resource originating from a company renowned for its significant place in criminal network history.

The once-dominant Enron Corporation, previously atop the energy, natural gas, and telecommunications sectors and praised for its innovation by Fortune magazine, fraught with crisis, succumbed to bankruptcy in a matter of weeks. Investigations have revealed widespread financial fraud, incriminating numerous executives in elaborate criminal undertakings [50]. The primary form of communication within Enron is via email; these archives became a key asset for investigative purposes, ultimately leading to the conviction of 28 executives, including a 120-year sentence for CEO Kenneth Lay. The investigative exposure of the Enron email dataset has galvanized analyses seeking to dissect criminal associations and network frameworks, along with the prospect of discerning suspects [51]. Because of the dataset’s direct linkage to criminal activities and its structurally intricate network, coupled with its ability to align experimental findings with authentic judicial outcomes, the SNFA model adopts the Enron email dataset for analysis. Table 1 lists the convicted Enron executives.

Comprising everyday communication among staff, the Enron email compilation was rife with anomalies and superfluity. Preprocessing efforts are vital for standardization, catering to the node vectorization of SNFA, which necessitates data in an encoded format. The encoding process transformed individuals within the dataset into a uniform node format, preparing the data for incorporation into network representation learning algorithms, culminating in three distinct preprocessed datasets: a communication edge table, a node table, and a node communication table, as shown in Table 2.

Table 2a outlines the communication edge table, where the first two columns catalog encoded identifiers for the sender and receiver nodes, respectively, and the third column quantifies the interaction strength between nodes, together constructing an elementary network graph [52]. Table 2b shows the corresponding pairs of node identities and their encrypting numbers, thus simplifying the node recognition. Table 2c tabulates, per node, the volumetric count of communications and the assortment of unique interconnected nodes, reflecting network activity and guiding the selection of analyzable nodes.

Although the Enron email dataset provides a valuable and detailed source of communication data relevant to criminal network analysis, it is crucial to acknowledge its limitations in terms of generalizability. The dataset is specific to a particular corporate environment and time period, which may not fully represent the dynamics of modern communication platforms such as social media or contemporary email systems. These differences can affect the applicability of the findings to broader contexts. Consequently, although the results obtained using the Enron dataset are insightful and demonstrate the effectiveness of the SNFA model, caution should be exercised when extending these findings to other settings. Modern social networks and communication platforms such as WeChat, Twitter, and others differ significantly in terms of user behavior, content types, and network structures. Therefore, the insights gained from the Enron dataset may not fully translate into these contemporary platforms. This limitation should be considered when interpreting results and assessing the broader applicability of the SNFA model.

### 3.2. Parameters Settings

The SNFA model utilizes three common evaluation metrics, Precision, Recall, and F-value, to assess the model’s accuracy and performance. Higher values in these metrics indicate a better fit and more accurate results, whereas lower values suggest a poorer fit. The concept of a Confusion Matrix is introduced, where rows represent actual categories and columns represent predicted categories, with cell values indicating the count of classified data points. As shown in Table 3, the Confusion Matrix uses T for positive classes, F for negative classes, P for positive instances, and N for negative instances. TP indicates true positives, TN true negatives, FP false positives, and FN false negatives.

Precision represents the probability that the true data are correctly identified as positive, as defined by:

(9) $$Precision=\frac{TP}{TP+FP}.$$

Recall quantifies the likelihood of accurately identifying positive instances in the original sample, as delineated in:

(10) $$Recall=\frac{TP}{TP+FN}.$$

The F-value, which harmonizes Precision and Recall to enhance experimental outcomes, is frequently regarded as the most critical metric, as represented by:

(11) $$F-value=2*\frac{Precision*Recall}{Precision+Recall}.$$

Given the SNFA model’s foundation in network representation learning, representing nodes as vectors containing information and network structure, choosing the appropriate parameters is crucial for accurate and efficient representation. The parameters of the Node2vec algorithm significantly affect the process and results. Controlled variable experiments were conducted to understand the influence of the individual parameters and their interactions. The initial parameters were set to t = 30, d = 128, W = 10, γ = 40, p = 2, and q = 0.5, focusing on the learning dimensions and sampling frequencies.

**Figure 4:**
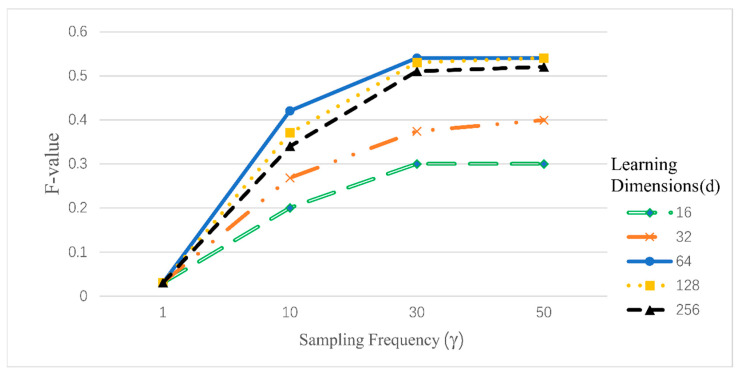
The F-measure under the changes of γ and d.

Figure 4 shows the impact of γ and d on the F-value, indicating that both the sampling frequency and learning dimensions significantly affect the results of the SNFA model. For sampling frequency γ, the results improved substantially with values from 1 to 10, moderately from 10 to 30, and plateaued beyond 30, suggesting an optimal range of 30 to 50 for balancing accuracy and computational efficiency. Learning dimensions of 64 or 128 yield better results, as too-low dimensions may lose information, while too-high dimensions can lead to redundancy, decreasing accuracy.

**Figure 5:**
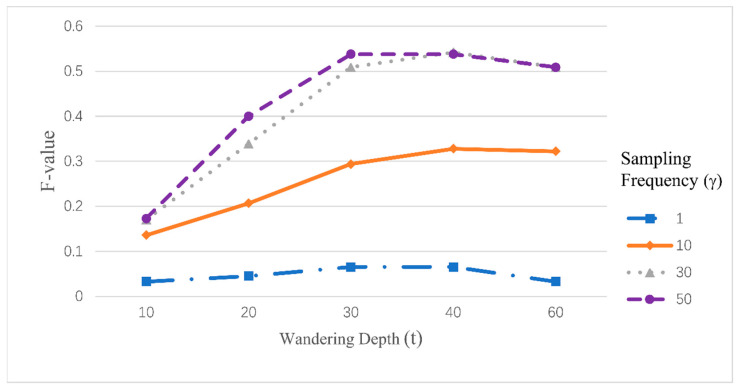
The F-measure under the changes of t and γ.

Figure 5 illustrates the effect of walk depth t and sampling frequency γ on the F-value, with similar patterns observed in sampling frequency, where increasing values improve the results up to a point. The choice of the walk depth t generally depends on the dataset size, with larger datasets requiring greater walking depths. A walk depth of 30 was chosen based on the data to optimize the experimental outcomes.

### 3.3. Results

The SNFA model employed network representation learning for social network forensics and was compared with conventional forensic methods, CrimeNet Explorer [53] and LogAnalysis [54], both of which utilize network topology approaches.

1. CrimeNet Explorer: This forensic method is specifically designed for criminal networks by segmenting a criminal network into multiple subnetworks. It measures using three centrality values and employs shortest path algorithms and Blockmodeling to determine the closeness between nodes in a criminal network, identifying core members and even top leaders.
2. LogAnalysis: This method automates the import of telephonic communication data by utilizing MCD data. It incorporates network topology, criminal investigation, and statistical analysis to establish a framework for revealing and analyzing the structure and behavior of criminal networks. Based on the Newman and GN algorithms, it measures using betweenness centrality and employs a greedy algorithm for hierarchical clustering, identifying closely connected clusters and core members through structural analysis.

**Figure 6:**
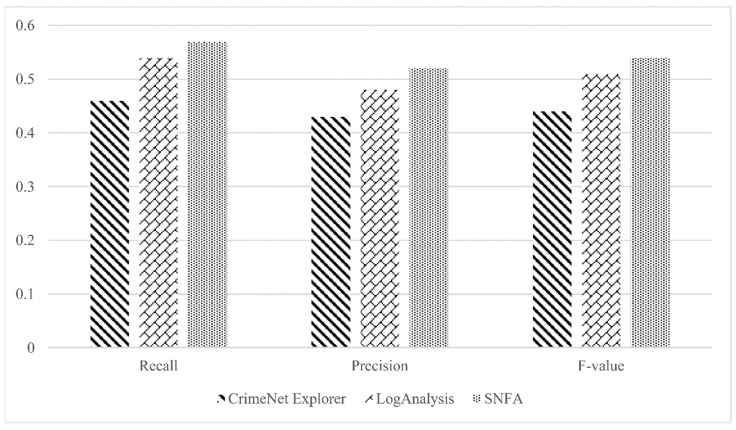
Comparison of the three forensic schemes.

Figure 6 and Table 4 present comparisons of the three-evaluation metrics across the three forensic approaches using the Enron dataset. The parameters for the Node2vec random walk were set to p = 2, q = 0.5, window size = 15, sampling frequency γ = 50, walk depth t = 40, and learning dimension d = 64.

The accompanying chart clearly demonstrates the superiority of the SNFA model over both LogAnalysis and CrimeNet Explorer across precision, recall, and F-value metrics, indicating a marked advancement over CrimeNet Explorer. These findings substantiate the efficacy of the SNFA model in pinpointing key individuals and leadership in criminal networks.

The SNFA model applies multidimensional data provided by the MCD for more effective data mining and analysis. The utilization of the Enron email dataset streamlines the construction and hierarchical examination of criminal networks. In contrast to traditional topological techniques, the SNFA model employs network representation learning to transform network nodes into vectors, thereby simplifying the assessment of the relationships between nodes. The integration of the Node2vec algorithm refines random walk strategies for the nuanced representation of non-adjacent node interactions, thus enhancing experimental precision. The SNFA model uses the CBOW and Hierarchical Softmax models for nodal vectorization by formulating rules to select computable nodes grounded in social network traits. This method not only boosts computational speed and analytic proficiency but also advances the gradient update process within the Huffman tree of the output layer. Hierarchical clustering was then used to determine the optimal cluster count (k-value), with subsequent iterative relocation to achieve the desired clustering. Ultimately, the efficacy of the SNFA model is both theoretically and empirically contrasted with traditional forensic approaches, namely CrimeNet Explorer and LogAnalysis. Outperforming these platforms in all three evaluation criteria, the SNFA enhancements are particularly noteworthy when juxtaposed with CrimeNet Explorer. Establishing its practicality, the SNFA model proved adept at uncovering central figures in criminal networks, possibly extending it to the top echelons of leadership.

## 4. Conclusions and Outlook

Owing to the limitations of traditional forensic algorithms based on web analysis and community structures, this study adopted network representation learning algorithms to implement a social network forensic model. It outlines the background, development, and current state of social network forensics research, introduces the basic concepts of forensics and fundamental network characteristics, and compares various classic network representation learning algorithms. The Node2vec algorithm was chosen to map the criminal networks into vector spaces for computational analysis. The model integrates the Continuous Bag-of-Words (CBOW) model with the Hierarchical Softmax acceleration algorithm to refine the node sampling process, making it more aligned with the hierarchical features of social networks. The SNFA model employs hierarchical clustering to achieve optimal clustering by identifying core nodes within the criminal network. The importance of these core nodes is determined through similarity calculations, potentially identifying high-level leaders or suspects and aiding law enforcement in dismantling criminal networks.

It should be noted that while the SNFA model demonstrated effectiveness when applied to the Enron email dataset, its application to modern social network apps or communication platforms, such as WeChat or Twitter, may encounter challenges. These platforms often contain a significant amount of irrelevant content and tenuous connections, which can hinder the model’s ability to accurately identify key figures within criminal networks. Future research should explore methods to filter and preprocess data from such platforms in order to enhance the applicability of the SNFA model in contemporary forensic investigations.

The main work of this paper includes:

1. The basic principles and technologies of social networks are analyzed to address the shortcomings of traditional forensic methods. This paper proposes the use of network representation learning to map criminal networks into a vector space, accurately representing node attributes and network structure and facilitating the calculation of inter-node relationships.
2. The CBOW and Hierarchical Softmax models were selected for network node sampling and encoding. Owing to the different levels of closeness between network nodes and the hierarchical nature of networks, the SNFA model refines the sampling process to make it more reasonable and improves the gradient calculation process in the Huffman tree of the output layer.
3. Using clustering to identify core nodes within the criminal network, we address the significant influence of the initial cluster center selection in traditional K-Means clustering. The SNFA model proposes an improved hierarchical clustering method coupled with iterative reallocation to achieve optimal clustering.
4. The Enron company crime dataset was chosen for its well-defined criminal network structure and available sentencing results for comparison. The SNFA model demonstrates improvements over the traditional forensic methods LogAnalysis and CrimeNet Explorer based on three evaluation metrics: Precision, Recall, and F-value.

Despite the SNFA model outperforming LogAnalysis and CrimeNet Explorer in the experiments, there is significant room for improvement. Future research should focus on the following aspects:

1. Exploring deep neural network-based representation learning algorithms or incorporating higher-order similarities to calculate relationships more accurately between non-adjacent nodes.
2. Improving the methodology for identifying core nodes and the formulas for calculating their similarity. Deep diving into the hierarchical clustering structure or considering other clustering methods might enhance the results.
3. Employing natural language processing to semantically analyze the Enron email dataset, potentially yielding more comprehensive and accurate results when combined with an analysis of the topological structure criminal network. Additionally, considering other crime datasets with more nodes could lead to more accurate experimental outcomes owing to more thorough random walks and sampling.

## Figures and Tables

**Table 1:** List of Enron’s criminal executives.

Name	Position
Andrew Fastow	CEO
Andrew Lewis	Director
Ben Glisan	Financial Director
Cliff Baxter	Vice President
Dan Bayly	COO
Davis Maxey	N/A
Hunter Shively	Vice President
Jeffery Skilling	CEO
Joe Hirko	Sub-Company CEO & Portland General Electric CEO
John Forney	Manager
John Lavorato	CEO
Kenneth Lay	CEO
Ken Rice	Enron broadband CEO
Kevin Hannon	non-Enron Employee
Kevin Howard	Vice President
Linda Lay	Kenneth Lay’s wife
Louis Borget	Oil & Gas CEO
Louise Kitchen	President
Mark Koenig	Vice CEO
Mark Taylor	Employee
Mike Krautz	CFO
Paula Rieker	Manager
Ray Bowen	Executive and CFO
Richard Causey	Chief Accountant
Richard Sanders	Vice Presidente
Rick Buy	Manager
Rod Hayslett	Vice President
Stephen Cooper	Interim CEO and CRO

**Table 2:** Experimental data preprocessing results: (**a**) the result of communication edge; (**b**) the result of node encoding; (**c**) the result of node communication.

2604	2605	1.0	sara.davidn	7448	1	3	1
1514	4537	20.0	trisha.hubbard	8300	2	6786	482
2676	409	3.0	schulmeyer.gerhard	7592	3	1	1
2114	409	2.0	ken.williams	5399	4	359	72
481	614	4.0	langfeldt.andrea	4430	5	2	1
3672	3673	1.0	anna.harris	5174	6	31	14
1208	3046	1.0	falbaum.william	5318	7	456	52
2203	2718	1.0	john.aleazurix	3472	8	2	2
1213	230	1.0	hunter.larry.jn	915	9	81	4
134	1932	1.0	hlopak.ed	4496	10	47	9
1079	3474	1.0	steve.whitaker	7417	11	307	17
3448	36	2.0	matthias.lee	695	12	89	21
3307	3890	3.0	emmons.suzette	5689	13	166	43
(**a**)			(**b**)		(**c**)		

**Table 3:** Confusion Matrix.

	**Predicted Outcome**	**Positive Case**	**Negative Case**
**Actual Condition**			
Positive Case		TP	TN
Negative Case		FP	FN

**Table 4:** Results of the three forensic approaches.

	CrimeNet Explorer	LogAnalysis	SNFA
Recall	0.46	0.54	0.57
Precision	0.43	0.48	0.52
F-value	0.44	0.51	0.54

## Data Availability

The data that support the findings of this study are openly available at https://www.cs.cmu.edu/~./enron/, accessed on 15 March 2024.
